# Slow and fast locomotor muscle adaptations to sustained intermittent hypoxaemia

**DOI:** 10.1016/j.jphyss.2026.100084

**Published:** 2026-06-20

**Authors:** Lise Paprzycki, Vincianne Jenart, Alexandre Legrand, Carine Michiels, Alexandra Tassin

**Affiliations:** a Laboratory of Respiratory Physiology, Pathophysiology and Rehabilitation, Research Institute for Health Sciences and Technology, University of Mons, Mons, Belgium; b Cell Biology Research Unit (URBC), Namur Research Institute for Life Sciences (NARILIS), University of Namur, Namur, Belgium

**Keywords:** Skeletal muscle, Myofibre types, Hypoxaemia, Oxygen

## Abstract

Episodic hypoxaemia is associated with muscle dysfunction in respiratory insufficiencies, but its effects depend on the hypoxia/reoxygenation pattern. Sustained Intermittent Hypoxemia (SIH) characterizes a large subgroup of COPD patients, yet muscle adaptations to SIH remain poorly known. We used a mouse model of SIH (FiO₂: 10%, 8 h/day) and analysed myofibre structure, muscle mass regulators, and myogenic markers in fast and slow-twitch hindlimb muscles. SIH induced an increase in haematocrit. At 35 days, *Soleus* cross-sectional area increased predominantly in slow-twitch fibres, but not in the fast *Tibialis Anterior (TA)* muscle. HIF1 target gene expression was increased at early timepoints with muscle-type-specific differences. While myostatin plasma levels were decreased, myostatin receptor and atrogene expression differed between muscles at baseline and upon SIH. *Myod1* and *Myog* expression decreased over time in the *TA* only*.* In conclusion, SIH induces muscle-type-specific adaptations, promoting hypertrophy in slow-twitch muscle while impairing myogenic regulation in fast-twitch muscle.

## Graphical Abstract

**Figure:**
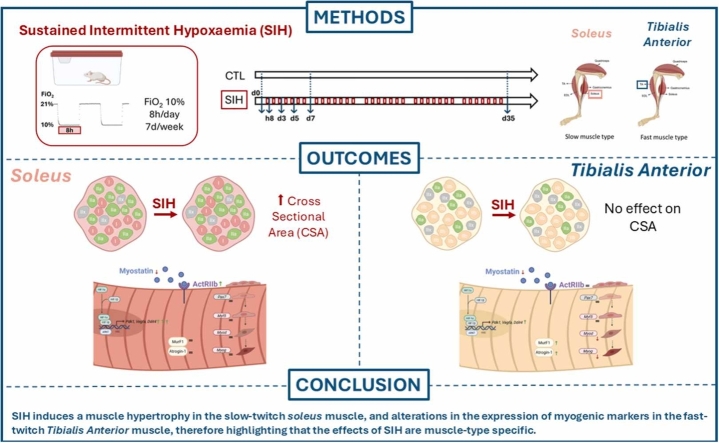

## Background

Respiratory insufficiencies represent a major public health issue due to their high frequency and severe impact on patient survival and quality of life [1], [2]. These diseases are associated with numerous comorbidities, including muscle dysfunction. The etiology of muscle dysfunction in respiratory diseases is multifactorial and notably includes genetic factors, systemic inflammation, medications, malnutrition, muscle deconditioning, and hypoxaemia [3], [4]. Hypoxaemia is a condition characterised by reduced haemoglobin oxygen saturation, leading to tissue hypoxia [5]. Because of confounding factors existing in clinical studies, animal models of hypoxaemia are used to decipher its muscle-specific effects.

Among respiratory insufficiencies, Chronic Obstructive Pulmonary Disease (COPD) is a frequent, poorly reversible respiratory disorder that is a leading cause of morbidity and mortality worldwide [3]. COPD includes chronic obstructive bronchiolitis and emphysema, leading to airflow obstruction, pulmonary hyperinflation, air trapping, and gas exchange abnormalities [6]. Numerous co-morbidities, including cardiovascular diseases, metabolic syndrome, osteoporosis, and muscle dysfunction, were reported in COPD [6]. It is worth mentioning that COPD-associated muscle dysfunction occurs both in respiratory and locomotor muscles and contributes to a reduction of exercise capacity. Reported molecular changes in locomotor muscles include oxidative stress, signs of structural damage, and an imbalance between proteolysis and protein synthesis [7]. A shift towards anaerobic pathways and a less fatigue-resistant phenotype were also described [8], [9].

Most animal studies aiming to decipher the effect of the hypoxaemic component of COPD use models of continuous exposure to hypoxaemia (24 h/24). However, in a large subgroup of patients with COPD, the progressive decline in respiratory function leads to hypoxaemia that is first characterised by episodes of nocturnal desaturations (affecting 38% of patients with moderate-to-severe COPD who do not qualify for home oxygen therapy based on daytime PaO₂) [10] and exercise-induced desaturations (61% of patients) before becoming persistent [11], [12]. Since the effect of this specific pattern is poorly known, our group has developed a murine model of Sustained Intermittent Hypoxaemia (SIH, 10% FiO₂, 8 h/24) to mimic moderate and episodic desaturations occurring in a large subgroup of COPD patients, as we described in [13]. With this pattern, our objective was to reproduce one component of an intermediate stage of the disease rather than advanced COPD. Indeed, at early stages, patients do not exhibit continuous hypoxaemia but instead experience intermittent desaturations, particularly during sleep or physical exercise (for example, when climbing stairs). An 8 h exposure to SIH during the light phase (resting phase in animals) was designed to reproduce a typical night of sleep, during which desaturation episodes are sustained and stable. To investigate the effect of hypoxaemia on skeletal muscle using this model and to limit biases, mouse movement restriction and excess dietary antioxidants must be avoided while maintaining a homogeneous FiO_2_ within the device. In line with these features, and even if the SIH model was not designed to capture the entire complexity of COPD pathophysiology, it has been specifically optimised to properly assess hypoxaemia-mediated skeletal muscle alterations.

Hypoxaemia results in cell adaptations to cope with the low oxygen availability [14]. When breathing room air (Fraction of inspired Oxygen (FiO₂) 21%, normoxia), oxygen partial pressure in resting muscles (physioxia) is around 40 mmHg or 5% atmospheric pressure. In this condition, the protein Hypoxia-Inducible Factor (HIF)−1α is constitutively synthesised but continuously degraded by the proteasome due to its hydroxylation and ubiquitination [15]. However, when oxygen partial pressure decreases, the HIF-1α protein is stabilised, it dimerises with the beta subunit, and the transcription factor HIF-1 acts as the master switch of the compensatory response to hypoxia, inducing the expression of genes such as *PDK1, DDIT4, VEGFA,* and genes encoding glycolysis enzymes [16]. Compensatory responses include Pyruvate Dehydrogenase Kinase 1 (PDK1)-mediated metabolic switch, Vascular Endothelial Growth Factor (VEGF)-mediated stimulation of angiogenesis, Erythropoietin (EPO)-mediated increased erythropoiesis and DNA Damage-Inducible Transcript 4 protein (DDIT4)-mediated cell growth arrest [15].

In healthy humans, the effect of hypoxaemia on skeletal muscle strongly depends on the depth, duration and pattern of hypoxia/reoxygenation phases (reviewed in [17]). For example, after acute exposure to high altitude (hypobaric hypoxaemia), muscle mass loss is associated with reduced Cross-Sectional Area (CSA) and reduced oxidative capacity of the *Vastus Lateralis* muscle [17], [18]. Severe and prolonged hypoxaemia (normobaric and hypobaric) has been shown to be detrimental for muscle function, resulting in impaired strength produced by locomotor muscles in response to sustained high-intensity exercise [19], [20]. The adaptation of locomotor muscles to hypoxia is often described as accompanied by an atrophy linked to a disruption of protein synthesis and degradation pathways [16], [17], [21]. The level of myostatin — a negative regulator of skeletal muscle mass — has been shown to be increased by hypoxia in both humans and mice in the plasma, and at the muscle mRNA level [22], [23]. However, studies conducted on murine models exposed to hypoxia revealed contradictory results depending on the experimental conditions, with effects that may be harmful or beneficial [24], [25]. As well, to the best of our knowledge, the specific effects of SIH at the muscular level have not been investigated yet.

Myofibre type composition is primordial in the investigation of hypoxemia consequences on locomotor muscle. Indeed, skeletal muscle myofibres can be classified as slow-twitch (type I), fast-twitch (type IIb) and intermediate (IIa and IIx) based on their contractile and metabolic profiles [26]. Sensitivity to hypoxia and hypoxia-mediated alterations are susceptible to differ according to myofibre type due to their differential metabolism and HIF-1α basal level [27]. When investigating the myofibre-type-specific effects of hypoxaemia, studies report different results depending on the pathological or physiological context, as well as the pattern of hypoxia episodes, as reviewed in [28]. Notably, a study exposing mice to continuous normobaric hypoxaemia (FiO₂: 8%) demonstrated a decrease in total CSA of the fast-twitch *EDL* (*Extensor Digitorum Longus*) muscle, primarily observed in type IIa and IIb fibres, but not in type IIx, suggesting fibre-type-specific effects [29]. In that study, the authors reported that hypoxia-mediated muscle atrophy was more prominent in the *EDL* than in the *Soleus* muscle and that their adaptive responses differed [29]. However, the impact of SIH according to fibre type has not yet been elucidated. On the other hand, myofibre composition is also adaptable in response to physiological and pathological conditions through a process known as fibre type switching, which is associated with modifications in both metabolic properties and myofibre size [28]. However, studies also report conflicting results regarding the mechanism of muscle fibre type switching, which is highly dependent on muscle types (fast/slow), myofibre composition [30] and the pattern of hypoxia exposure (acute/chronic, normobaric/hypobaric hypoxia, duration, depth, age of animals) [31], [32], [33]. In COPD patients, fibre type switching is also observed and depends on the stage of disease progression [34]. Reported effects highlighted that atrophy is associated with a switch from a slow to a fast phenotype [35], [36]. However, studies on COPD patients include confounding factors such as inflammation, age, and sedentary lifestyle, which complicate data interpretation regarding the specific effects of hypoxemia [34].

Hypoxaemia was also described to induce alterations in muscle regenerative potential ([37], [38], [39], reviewed in [40]), a process involving Satellite Cells (SCs). SCs are resident adult “stem” cells located between the sarcolemma and the basal lamina of myofibres. These cells are the main mediators of muscle regeneration [41], [42]. Indeed, quiescent SCs express Paired-box transcription factor 7 (*Pax7*). Once activated, a SC divides asymmetrically: **(i)** one daughter cell expresses *Myod1* (encoding the myogenic factor Myoblast determination protein 1) and differentiates into a myoblast, which proliferates, yielding myocytes that fuse with a pre-existing fibre; **(ii)** the other daughter cell loses *Myod1* expression and returns to a quiescent-like state to mediate SC self-renewal. As most stem cells, SCs reside in a hypoxic niche (reviewed in [41]). This microenvironment favours SC self-renewal and maintenance in an undifferentiated state by mechanisms involving O_2_-sensitive pathways (HIF1, NOTCH, WNT). In muscle stem cells, HIF-1 controls pluripotency gene expression, promotes glycolytic metabolism, and inhibits mitochondrial biogenesis and myogenic factor expression [43], [44]. In contrast, stem cell differentiation is associated with HIF-1α degradation [45] and a metabolic shift towards increased oxidative phosphorylation (OxPhos) (reviewed in [46]). The impact of hypoxaemia on muscle regeneration potential is still poorly understood, especially when considering the sustained intermittent pattern observed in many COPD patients.

In the present study, we aimed to decipher whether SIH induces muscle-specific alterations depending on muscle type and fibre composition. We used our SIH model to delineate the alterations induced by SIH in two locomotor muscles with different phenotypes: the slow-twitch *Soleus* muscle and the fast-twitch *Tibialis Anterior* (*TA*) muscle. We hypothesised that SIH could differentially alter those muscles. To further understand the mechanisms underlying these alterations, we investigated several regulators of muscle fibre differentiation and fibre type switching. This approach will lead to a better understanding of the specific effect of hypoxia on muscle alterations observed in hypoxemic COPD patients.

## Materials and methods

### Ethics statement

All procedures met the Belgian national standard requirements regarding animal care and were conducted in accordance with the Ethics and Welfare Committee of the University of Mons (reference number of the approved protocol: LE021/03).

### Animals

Male C57BL/6 mice were purchased from Charles River Laboratories (France). Mice were housed in a conventional animal facility in cages with *ad libitum* access to water and food. Mice were maintained at a 35–40% relative humidity and a constant room temperature (RT) of 21°C in a natural 12 h/12 h light–dark cycle. All experimental procedures were performed so that all mice were 14 weeks old at the time of sacrifice. Two weeks before the beginning of the experiment, mice were fed using a specific diet (D10012M AIN-93M Mature Rodent Diet, Research Diet) containing the recommended amounts of antioxidants (tert-butylhydroquinine tBHQ 0.01 g per 1000 g of weight, Mineral mix S10022M containing Zinc carbonate (52,1% Zn), Cupric Carbonate (57,5% Cu) and Sodium selenate (41,8% Se) and Vitamin mix V10037 containing Vitamin C and Vitamin E acetate). This food was used to ensure that hypoxaemia-related effects were not masked by dietary antioxidants, this point being particularly important as mice are inherently more resistant to oxidative stress due to their endogenous vitamin C production. Animals were subjected to an acclimatisation to the experimental room for a period of 7 days. At the beginning of the protocol, mice were randomly assigned to two experimental groups: mice submitted to sustained intermittent hypoxaemia (SIH, n = 5–12) and control mice (CTL, n = 4–12). SIH mice were exposed to SIH (10% FiO₂, 8 h/day, 7 d/week during light cycle) for 1 h, 8 h, 3, 5, 7, or 35 days in a device previously developed and validated [47]. This device was optimised to avoid movement restrictions using larger cages (39 ×25 x 15 cm) where oxygen flow was maintained uniformly across all areas of the cage (Supplemental Figure S1). CTL mice were exposed to ambient air in an identical cage and placed near the hypoxic device housing SIH mice to reproduce similar noises. Body weight and food intake were monitored every day from the acclimatisation period to the end of the protocol. The day following the end of the protocol, mice were sacrificed, and the slow-twitch *Soleus* muscles, the fast-twitch *Tibialis Anterior* (*TA*) muscles, blood, and tissues were collected for immunofluorescence labelling, RT-qPCR, and ELISA analyses. Haematocrit measurement was performed in the blood samples by using a haemocytometer.

### Tissue preparation and immunofluorescence labelling

*TA* and *Soleus* muscles were embedded in OCT (Optimal Cutting Temperature) compound (VWR International) and frozen in liquid-nitrogen-cooled isopentane. Cryostat sections (8 µm thick) were prepared using a Leica cryotome. Entire transverse muscle cryosections were blocked with 10% Goat-serum/PBS (VWR, S2000–100) for 1 h at RT. Cryosections were then incubated for 2 h at RT with the following antibodies in blocking solution: Myosin Heavy Chain 7 (MyHC7) (type I fibres, IgG2b, clone BA-D5, 1:50, DSHB, RRID: AB_2235587), MyHC2 (type IIa fibres, IgG1, clone SC-71, 1:100, DSHB, RRID: AB_2147165), MyHC4 (type IIb fibres, IgM, clone BF-F3, 1:10, DSHB, RRID: AB_2266724), MyHC1 (type IIx fibres, IgM, clone 6H1-Xi, 1:10, DSHB, RRID: AB_3696610) and laminin (rabbit IgG, ab 11,575, 1:50, Abcam, RRID: AB_298179). Slides were washed 3 times in PBS and incubated for 1 h with secondary antibodies directed against mouse IgG2b (Alexa 647 anti-mouse IgG2b, A-21242, 1:100, Thermofisher), mouse IgG1 (Alexa 488, anti-mouse IgG2b, A-21121, 1:100, Thermofisher), mouse IgM (Alexa 555, anti-mouse IgM, A-21426, 1:50, Thermofisher), and rabbit IgG (Alexa 405 anti-rabbit IgM, ab17652, 1:50, Abcam) to label respectively type I (Cy5 channel), type IIa (FITC channel), and type IIb/IIx (TRITC channel) myofibres as well as laminin (DAPI channel). Slides were washed 3 times in PBS and mounted with ProLong™ Gold Antifade Mountant (P36934, Invitrogen). Whole muscle images were taken using the CellDiscoverer 7 (10x magnification, Zeiss, Morph-Im Platform, UNamur).

### Image processing and measurements

Images were analysed using the ImageJ software. Cross-sectional area (CSA) of each myofibre was assessed manually using the Freehand ROI tool. The area measured in pixels was then converted into µm² using the image calibration settings. Myofibres were then classified into clusters according to their area (<500 μm² and every 500 μm² until 4500 μm² for the *Soleus* muscle and <1000 μm² and every 1000 μm² until 8000 μm² for the *TA* muscle) to evaluate changes in myofibre CSA distribution. CSA and distribution analysis were performed on muscle sections before performing fibre-type-specific analyses.

### RNA extraction – reverse transcription and real-time PCR

The total RNA from frozen *TA* and *Soleus* muscles was extracted using the Trizol reagent (Invitrogen) according to the manufacturer’s instructions. A DNAse I (amplification grade, Thermo Fisher Scientific) treatment of RNA was then performed. The retrotranscription was performed using 500 ng of RNA that were reverse transcribed into cDNA using the Maxima First Strand cDNA Synthesis Kit (Thermo Fisher Scientific). All qPCR reactions were performed with Lightcycler 480 Real-Time PCR II (F. Hoffmann Roche R ©, Ltd., Basel, Switzerland) using SYBR Green FastStart Essential DNA Green Master (Roche, Basel, Switzerland) and corresponding primers (Eurogentec, Seraing, Belgium) (Supplemental Table S2). Cycling conditions were as follows: initial denaturation step at 95 °C for 10 min, followed by 40 cycles of 15 s at 95 °C and 60 s at primer Tm. qPCR results were analysed with LightCycler 96 software (Roche). The gene cycle threshold (Ct) of the gene of interest was normalised to the expression of the housekeeping gene *Rplp0*, and gene expression was calculated using the 2^−∆∆Ct^ method.

### ELISA

Myostatin plasmatic concentrations were measured using the Human, Mouse, & Rat GDF−8/Myostatin Quantikine ELISA Kit according to the manufacturer’s instructions (DGDF80, R&D Systems, Minneapolis, MN, United States).

### Statistical analyses

Statistical analyses were performed using SigmaPlot software, version 14. For comparison, depending on normality and equal variance tests, we used: (i) Two-Way ANOVA Repeated Measure (body weight evolution), (ii) Two-Way ANOVA Analysis of Variance, Holm-Sidak method (haematocrit level, myostatin plasmatic level), (iii) Student’s *t*-test (myofibre CSA), (iv) Chi-square test (myofibre CSA distribution in the *Soleus* and *TA* muscles), (v) Mann-Whitney Rank Sum test (*Mstn, Acvr2b* and *Fbxo32* mRNA for *TA* vs *Soleus* analysis) or (vi) Kruskal-Wallis One-Way Analysis of Variance on Ranks (*Pdk1, Ddit4, Vegfa, Mstn, Acrv2b, Fbxo32, Trim63, Pax7, Myf5, Myod1 and Myog* mRNA). Differences were considered statistically significant for a p-value < 0.05. According to normality and equal variance test results, the graphical representations were performed as follows. Haematocrit, CSA, myofibre area distributions, and myostatin plasmatic level were expressed as mean ± SEM and presented as histograms. Myofibre type proportions were represented as stacked histograms. *Pdk1, Ddit4, Vegfa, Mstn, Acrv2b, Fbxo32, Trim63, Pax7, Myf5, Myod1*, and *Myog* mRNA levels were represented as boxplots (median, 25th and 75th percentiles).

## Results

### SIH induced a secondary erythrocytosis

**Fig. 1:**
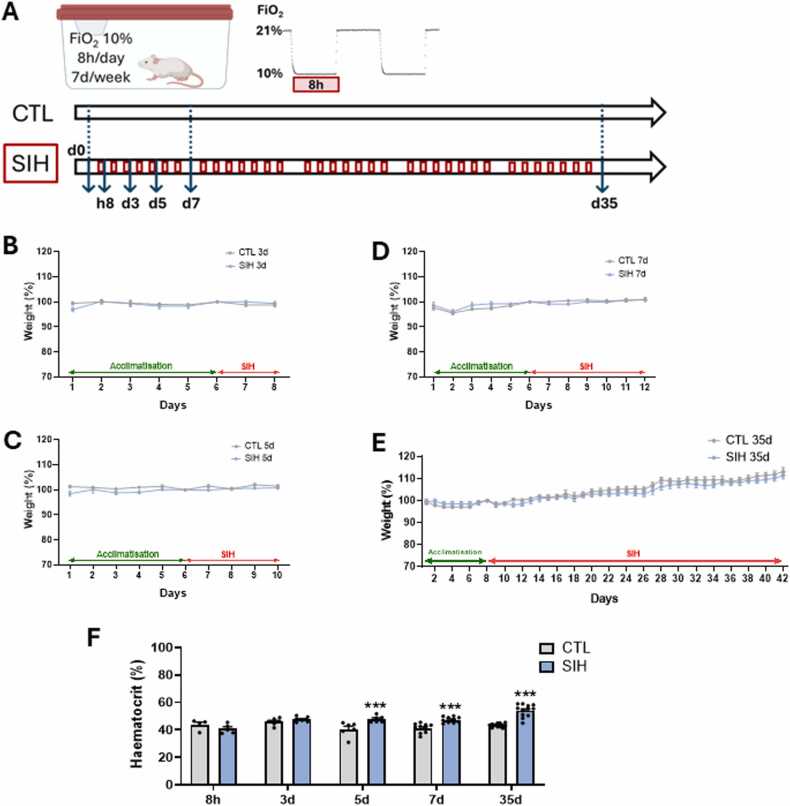
SIH exposure timeline and model validation. (A) Mice were exposed to SIH for 8 h, 3, 5, 7 or 35 days. (B) Effect of SIH on mouse body weight for 3 days; Two-Way ANOVA Repeated Measures: NS, SIH vs CTL (n = 6 for CTL, n = 6 for SIH). (C) Effect of SIH on mouse body weight for 5 days; Two-Way ANOVA Repeated Measures: NS, SIH vs CTL (n = 6 for CTL, n = 6 for SIH). (D) Effect of SIH on mouse body weight for 7 days; Two-Way ANOVA Repeated Measures: NS, SIH vs CTL (n = 10 for CTL, n = 10 for SIH). (E) Effect of SIH on mouse body weight for 35 days; Two-Way ANOVA Repeated Measures: NS, SIH vs CTL (n = 12 for CTL, n = 12 for SIH). (F) Haematocrit level. Data represented as mean ± SEM; Two-Way ANOVA: ***: p < 0.001, SIH vs CTL (n = 4–12 for CTL, n = 5–12 for SIH).

To decipher the specific effect of episodic hypoxaemia on skeletal muscle, we used a mouse model of Sustained Intermittent Hypoxaemia (SIH). Mice were exposed to a FiO₂ of 10% (8 h/day) for 8 h, 3, 5, 7, and 35 days, in a device optimised to avoid movement restriction and to ensure a homogeneous distribution of gas flow. Control mice (CTL) were kept in a similar device while exposed to ambient air (FiO_2_: 21%) (Fig. 1A). Body weight was measured daily throughout the whole protocol. The results showed that SIH did not affect this parameter when compared to the CTL group (Fig. 1B-E). Hypoxaemia is known to induce a secondary erythrocytosis [48]. As a control of tissue compensatory response to hypoxia, haematocrit was measured directly after sampling. From 5 days of exposure, the haematocrit was increased in the mice of the SIH group as compared to CTL mice (Fig. 1F).

### SIH induced a type-I fibre hypertrophy in the *Soleus* muscle

**Fig. 2:**
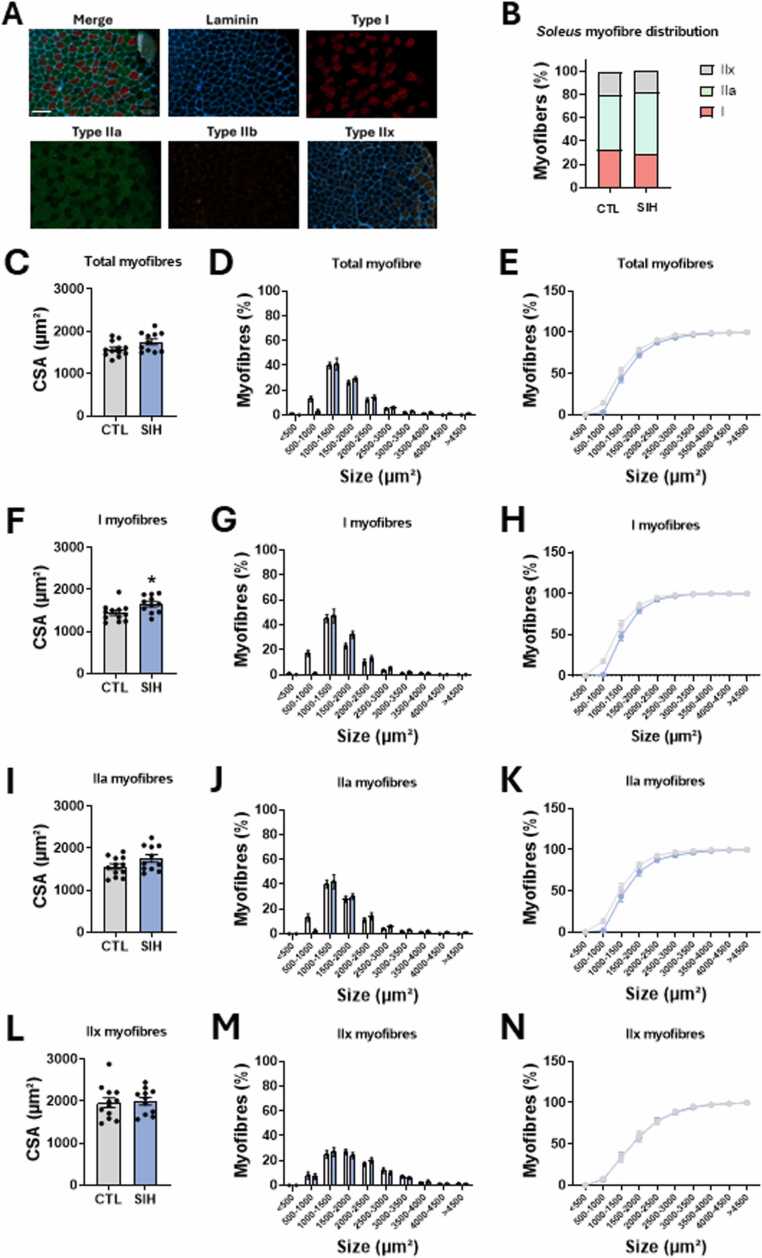
Effects of 35 days of SIH on mouse *Soleus* muscle (Cross-Sectional Area (CSA) and myofibre size distribution). (A) *Soleus* muscle cryosections of SIH and CTL mice were labelled using antibodies directed against MyHC7 (type I fibres), MyHC2 (type IIa fibres), MyHC4 (type IIb fibres), MyHC1 (type IIx fibres) or laminin. Scale bar = 100 µm. (B) Myofibre type proportions. Data presented as stacked bars; groups compared using a *t*-test: NS. (C, F, I, L) Each myofibre CSA was measured using the ImageJ software. Data represented as mean ± SEM. (D, G, J, M) Myofibres were classified in clusters according to their area (µm²). (E, H, K, N) Cumulative percentage. (C) CSA on the whole *Soleus* muscle; *t*-test: p = 0.056 (n = 11 for CTL, n = 10 for SIH). (D, E) Whole *Soleus* fibre size distribution; Chi²: NS (n = 11 for CTL, n = 10 for SIH). (F) *Soleus* type I myofibre CSA; *t*-test: *: p < 0.05, SIH vs CTL (n = 11 for CTL, n = 10 for SIH). (G, H) *Soleus* type I fibre size distribution; Chi²: ***: p < 0.001, SIH vs CTL (n = 11 for CTL, n = 10 for SIH). (I) *Soleus* type IIa myofibre CSA; *t*-test: NS (n = 11 for CTL, n = 10 for SIH). (J, K) *Soleus* type IIa fibre size distribution; Chi²: NS (n = 11 for CTL, n = 10 for SIH). (L) *Soleus* type IIx myofibre CSA; *t*-test: NS (n = 11 for CTL, n = 10 for SIH). (M, N) *Soleus* type IIx fibre size distribution; Chi²: NS (n = 11 for CTL, n = 10 for SIH).

Since hypoxaemia is reported to induce a myofibre type switch [49], fibre type composition was assessed after 35 days of exposure to SIH. As expected, type I, type IIa and type IIx myofibres were detected in CTL and SIH slow‑twitch *Soleus* muscles (Fig. 2A). In the CTL group, the percentages of type I, type IIa and type IIx myofibres in the *Soleus* muscle were 32.7 ± 1.8%; 47.4 ± 1.8%, and 19.8 ± 3%, respectively. These proportions were not significantly modified upon SIH (Fig. 2B). We then assessed the impact of SIH on the Cross-Sectional Areas (CSA) of *Soleus* muscle fibres. Considering all *Soleus* myofibres, muscle fibre CSA (Fig. 2C) and myofibre CSA distribution (Fig. 2D-E) were not significantly different in mice submitted to SIH as compared to control mice. However, considering myofibre-type-specific analyses, SIH induced a higher type I fibre CSA compared to the CTL group, with a mean of 1443.4 ± 201.5 µm² for the CTL and 1654.6 ± 204 µm² for the SIH group (Fig. 2F). This result was also evidenced in the CSA distribution (Fig. 2G) and cumulative percentage (Fig. 2H) graphs. Indeed, there were more fibres in smaller area clusters in the CTL group (500–1000 µm²) and more fibres in higher area clusters in the SIH group (1500–3000 µm²). No change was observed in IIa and IIx myofibre CSA when mice were submitted to SIH (Fig. 2I-K and Fig. 2L-N, respectively). Taken together, these results highlight that SIH has a weak hypertrophic effect on the *Soleus* muscle, mostly affecting type I myofibres.

### SIH induced no morphometrical modification in the *TA* muscle

**Fig. 3:**
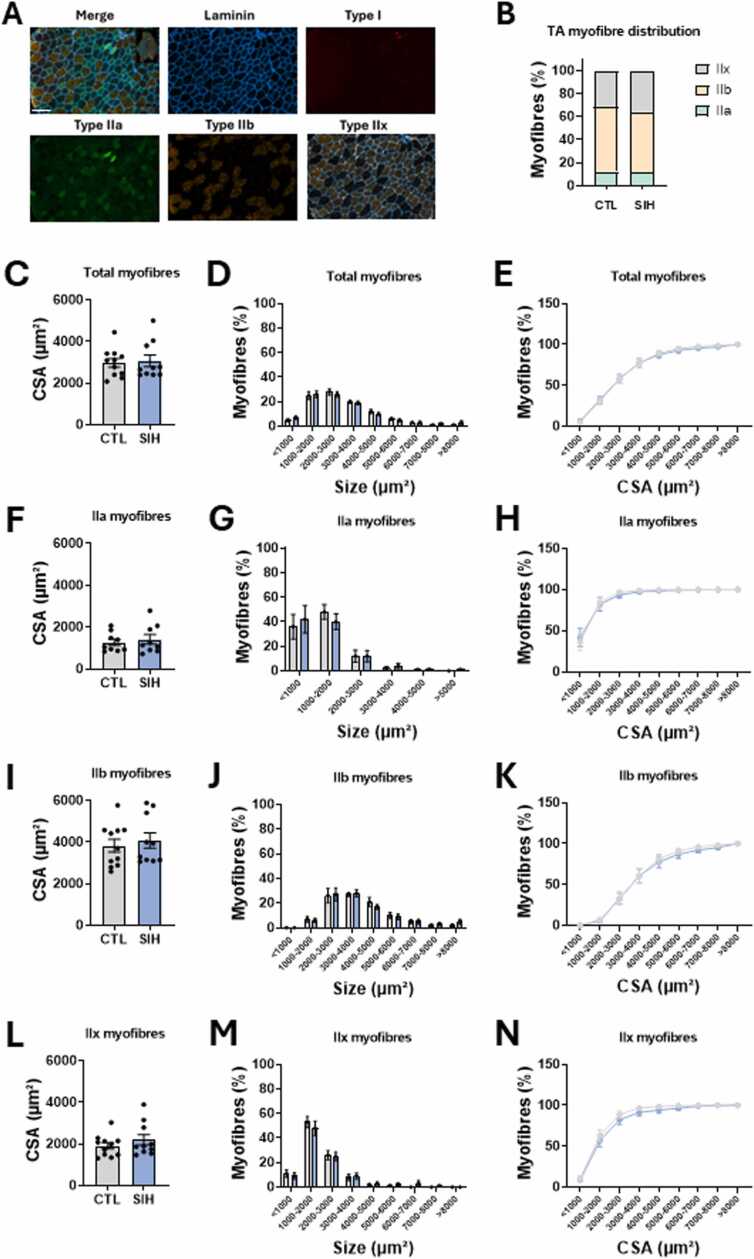
Effects of 35 days of SIH on the mouse *tibialis anterior* (*TA*) muscle (Cross-Sectional Area (CSA) and myofibre size distribution). (A) *TA* muscle cryosections of SIH and CTL mice were labelled using antibodies directed against MyHC7 (type I fibres), MyHC2 (type IIa fibres), MyHC4 (type IIb fibres), MyHC1 (type IIx fibres), or laminin. Scale bar = 100 µm. (B) Myofibre type proportions. Data presented as stacked bars; groups compared using a *t*-test: NS. (C, F, I, L) Each myofibre CSA was measured using the ImageJ software. Data represented as mean ± SEM. (D, G, J, M) Myofibres were classified in clusters according to their area (µm²). (E, H, K, N) Cumulative percentage. (C) CSA on the whole *TA* muscle section; *t*-test: NS (n = 11 for CTL, n = 10 for SIH). (D, E) Whole *TA* fibre size distribution; Chi²: NS (n = 11 for CTL, n = 10 for SIH). (F) *TA* type IIa myofibre CSA; *t*-test: NS (n = 11 for CTL, n = 10 for SIH). (G, H) *TA* type IIa fibre size distribution; Chi²: NS (n = 11 for CTL, n = 10 for SIH). (I) *TA* type IIb myofibre CSA; *t*-test: NS (n = 11 for CTL, n = 10 for SIH). (J, K) *TA* type IIb fibre size distribution; Chi²: NS (n = 11 for CTL, n = 10 for SIH). (L) *TA* type IIx myofibre CSA; *t*-test: NS (n = 11 for CTL, n = 10 for SIH). (M, N) *TA* type IIx fibre size distribution; Chi²: NS, (n = 11 for CTL, n = 10 for SIH).

To assess whether the impact of SIH depends on muscle type, morphometrical analyses were also performed on the fast *Tibialis Anterior* (*TA*) muscle after 35 days of SIH. As expected, type IIa, type IIb and type IIx fibres were detected in the *TA* muscle (Fig. 3A). In the CTL group, the percentages of type IIa, type IIb, and type IIx myofibres were 12.2 ± 3.6%; 56.5 ± 4%, and 31.3 ± 2.5%, respectively. These proportions were not significantly modified upon SIH (Fig. 3B). SIH did not impact either the mean CSA (Fig. 3C) or distribution (Fig. 3D-E) of the whole *TA* muscle when compared to the CTL group. The fibre-type specific analyses (IIa: Fig. 3F-H; IIb: Fig. 3I-K; and IIx; Fig. 3L-N) showed no effect of SIH on mean CSA or myofibre distribution. These results suggest that SIH did not impact the *TA* myofibre CSA after 35 days of exposure.

### SIH induced HIF-1 target gene expression in *Soleus* and *TA* muscles

The hypoxia-inducible factor−1α (HIF-1α) is a protein stabilised in low oxygen concentrations. Upon dimerization with HIF-1ß, it forms the transcription factor HIF-1, which acts as the master switch of the compensatory response to hypoxia [15]. HIF-1 induces the transcription of target genes involved in tissue metabolic adaptation to the lack of oxygen, notably *Pdk1*, *Ddit4* and *Vegfa* [50], [51]. We thus evaluated the mRNA levels of *Pdk1, Ddit4,* and *Vegfa* at early timepoints of exposure to SIH. For this analysis, an additional group of mice exposed to 1 h of hypoxia was included to assess the early transcriptional activation of HIF-1 target genes in response to SIH.

**Fig. 4:**
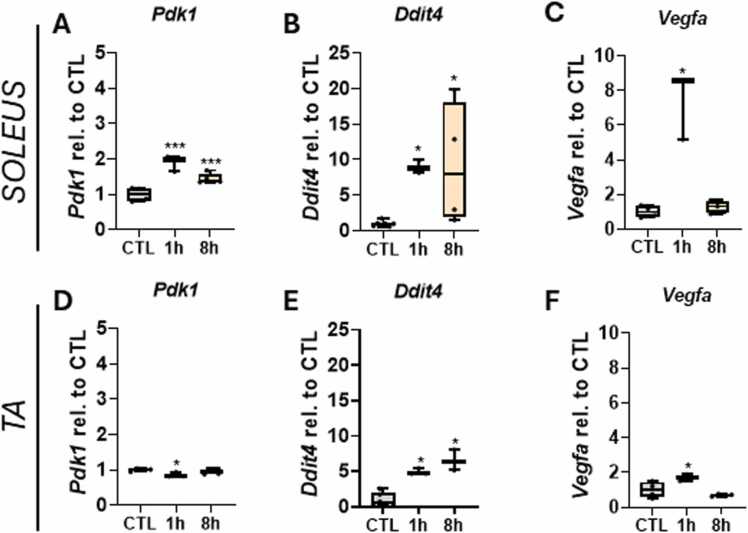
Effects of SIH exposure over time on the expression of HIF-1 target genes and modulators of cellular response to hypoxia in the *Soleus* (A-C) and the *TA* (D-F) muscles. (A-C) RT-qPCR were performed on the *Soleus* muscle, and the expression of several genes was quantified by using the ΔΔCt method (housekeeping gene: *Rplp0*; data normalized to CTL). (A) *Pdk1* (encoding PDK1) expression; One-Way ANOVA: ***: p < 0.001, 1 h and 8 h vs CTL (n = 4 for CTL, n = 3 for 1 h, n = 5 for 8 h). (B) *Ddit4 (*encoding REDD1) expression; One-Way ANOVA: *: p < 0.05, 8 h vs CTL (n = 4 for CTL, n = 3 for 1 h, n = 5 for 8 h). (C) *Vegfa* (encoding VEGF) expression; One-Way ANOVA: *: p < 0.05, 1 h vs CTL (n = 4 for CTL, n = 3 for 1 h, n = 5 for 8 h). (D-F) RT-qPCR were performed on the *Tibialis Anterior* (*TA)* muscle, and the expression of several genes was expressed by using the ΔΔCt method (housekeeping gene: *Rplp0*; data normalised to CTL). (D) *Pdk1* (encoding PDK1) expression; One-Way ANOVA: *: p < 0.05, 1 h vs CTL (n = 4 for CTL, n = 3 for 1 h, n = 4 for 8 h). (E) *Ddit4* (encoding REDD1) expression; One*-*Way ANOVA: *: p < 0.05, 8 h vs CTL (n = 4 for CTL, n = 3 for 1 h, n = 4 for 8 h). (F) *Vegfa* (encoding VEGF) expression; One-Way ANOVA: *: p < 0.05, 8 h vs 1 h (n = 4 for CTL, n = 3 for 1 h, n = 4 for 8 h).

In the *Soleus* muscle, we observed an increased *Pdk1* (encoding PDK1) gene expression at both 1 h and 8 h of exposure to SIH (Fig. 4A). Regarding *Ddit4* (encoding REDD1) gene expression, it was increased in the *Soleus* muscle at both 1 h and 8 h of exposure to SIH (Fig. 4B). *Vegfa* (encoding VEGF) gene expression was enhanced after 1 h of SIH (Fig. 4C), then it returned to the basal level.

In the *TA* muscle, we observed a decreased *Pdk1* gene expression after 1 h of exposure to SIH (Fig. 4D), which was no longer detected at 8 h. *Ddit4* gene expression was increased upon SIH after 1 h and 8 h of exposure (Fig. 4E). Regarding *Vegfa* gene expression, there was a marked increase in its expression at 1 h (Fig. 4F), then it returned to basal level.

At later time points, no change was observed in either of the two muscles regarding the expression of these genes upon SIH (Supplemental Fig. S4).

These results show a strong increase in the expression of HIF-1 target genes after acute exposure to SIH, particularly marked in the slow *Soleus* muscle. This increase was also observed in the *TA* muscle*,* but to a lower extent.

### SIH induced alterations in the expression of protein degradation modulators

Myostatin is a major regulator of muscle mass, favouring skeletal muscle wasting [52]. Since we observed a modification of type I fibre CSA in the *Soleus* muscle, we assessed the expression of *Mstn* (encoding Myostatin) and its receptor, *Acrv2b* (encoding ActRIIB).

**Fig. 5:**
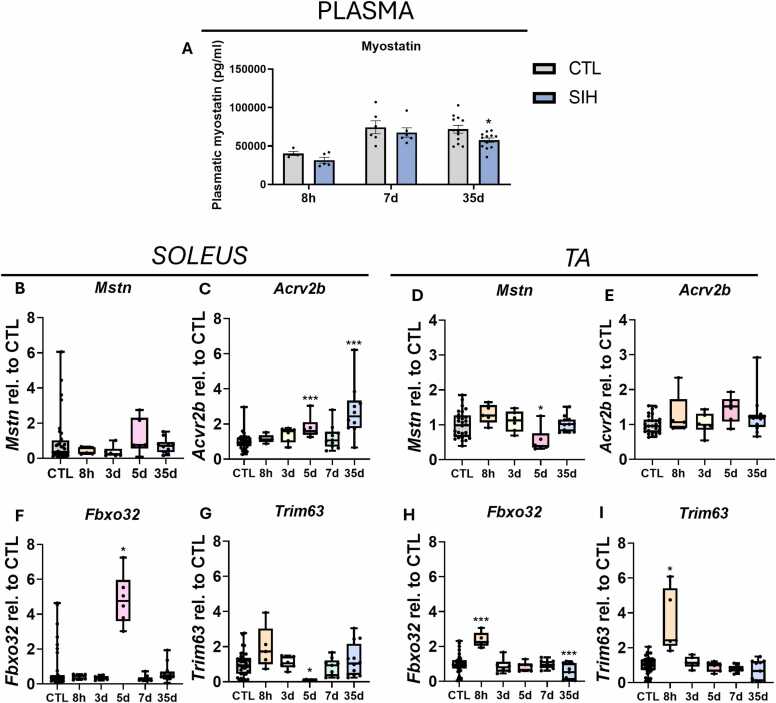
Effects of SIH exposure over time on the expression of protein degradation regulators in plasma, *Soleus,* and *TA* muscles. (A) Plasma myostatin levels measured by ELISA. Data presented as Mean ± SEM. Two-Way ANOVA:, p < 0.01, 8 h vs 7d and 35d, *p < 0.05, SIH 35d vs CTL 35d (CTL: n = 12; SIH: n = 12). (B-I) RT-qPCR were performed on the *Soleus* and *TA* muscles. Gene expression was analysed by using the ΔΔCt method (housekeeping gene: *Rplp0*; data normalised to CTL). (B) *Mstn* (encoding Myostatin) expression in the *Soleus* muscle; One-Way ANOVA: NS (CTL: n = 4–12; SIH: n = 5–12). (C) *Acvr2b* (encoding ActRIIB) expression in the *Soleus* muscle; One-Way ANOVA: ***: p < 0.001, 5d and 35d vs CTL (CTL: n = 4–12; SIH: n = 5–12). (D) *Mstn* (encoding Myostatin) expression in the *TA* muscle; One-Way ANOVA: *: p < 0.05, 5d vs CTL (CTL: n = 4–12; SIH: n = 5–12). (E) *Acvr2b* (encoding ActRIIB) expression in the *TA* muscle; One Way ANOVA: NS (CTL: n = 4–12; SIH: n = 5–12). (F) *Fbxo32* (encoding Atrogin−1) expression in the *Soleus* muscle; One-Way ANOVA: *: p < 0.05, 5d vs CTL (CTL: n = 4–12; SIH: n = 5–12). (G) *Trim63* (encoding MuRF1) expression in the *Soleus* muscle; One-Way ANOVA: *: p < 0.05, 5d vs CTL (CTL: n = 4–12; SIH: n = 5–12). (H) *Fbxo32* (encoding Atrogin−1) expression in the TA muscle; One-Way ANOVA: ***: p < 0.001, 8 h and 35d vs CTL (CTL: n = 4–12; SIH: n = 5–12). (I) *Trim63* (encoding MuRF1) expression in the *TA* muscle; One-Way ANOVA: *: p < 0.05, 8 h vs CTL (CTL: n = 4–12; SIH: n = 5–12).

We first compared the *Mstn* (encoding Myostatin) and *Acrv2b* (encoding ActRIIB) expressions in both *TA* and *Soleus* in CTL mice to better delineate possible differential expression of the two genes in both muscles at the basal level. *Mstn* expression was 27-fold higher in *TA* muscle when compared to the *Soleus* muscle (Supplemental Fig. S5A). Upon 35 days of SIH exposure, the myostatin expression was still higher in the *TA* muscle than in the *Soleus* muscle (Supplemental Fig. S5C). The gene encoding the myostatin receptor, *Acrv2b*, exhibited a similar expression in the two muscles (Supplemental Fig. S5B). Interestingly, when mice were submitted to SIH, *Acrv2b* was more expressed in the *Soleus* muscle compared to the *TA* muscle at 35 days (Supplemental Fig. S5D). However, myostatin plasma level decreased in SIH mice at 35 days of SIH (65445.6 ± 1371.7 µg/ml) compared to CTL mice (85345.6 ± 4965.4 µg/ml) (Fig. 5A). We also noticed an increased myostatin plasmatic level with time, regardless of whether the mice were exposed or not to SIH (ANOVA Two-Way: p < 0.01; Fig. 5A). We then assessed the muscle-specific effects of SIH on *Mstn* and *Acrv2b* at 8 h as well as after 1, 3, 5, 7, and 35 days of exposure (Fig. 5B-E). In the *Soleus* muscle, SIH did not affect myostatin expression (Fig. 5B). However, *Acrv2b* expression was increased both at 5 days and 35 days of exposure to SIH (Fig. 5C). On the other hand, *TA* myostatin expression was decreased at 5 days of SIH (Fig. 5D) with no effect observed on *Acrv2b* (Fig. 5E). Taken together, we observed differential responses to SIH depending on the muscle of interest. In particular, *Acrv2b* expression was upregulated in the *Soleus* muscle, whereas *Mstn* expression was slightly decreased in the *TA* muscle. It should also be noted that these two muscles display very different expression levels for these two genes.

We also investigated the expression of two atrogenes encoding muscle-specific ubiquitin-ligases: *Fbxo32* (encoding Atrogin−1) and *Trim63* (encoding MuRF1). First, basal expression of *Fbxo32* did not differ between *TA* and *Soleus* muscles in CTL mice (Supplemental Fig. S5E). Interestingly, *Fbxo32* was less expressed in the *Soleus* when compared to *TA* after 8 h of SIH (Supplemental Fig. S5F). This difference was no longer observed at 35 days of SIH (Supplemental Fig. S5G). We then assessed the muscle-specific effect of SIH on atrogene expression over time (Fig. 5F-I). In the *Soleus* muscle, SIH increased *Fbxo32* expression, but only at 5 days of SIH (Fig. 5F). On the contrary, at the same time point, *Trim63* expression was decreased in SIH mice (Fig. 5G). Regarding the *TA* muscle, *Fbxo32* expression increased after 8 h of exposure to SIH, then returned to the basal level and decreased at 35 days (Fig. 5H). *Trim63* expression was also increased at 8 h of SIH when compared to CTL mice and then returned to basal level at later time points (Fig. 5I). Taken together, the kinetics of atrogene expression differed among the muscles of interest, with later changes in the *Soleus* and earlier changes in the *TA* muscle. Furthermore, *Fbxo32* exhibited lower expression in the *Soleus* compared to the *TA* when the mice were exposed to acute SIH.

### SIH induced myogenic factor expression alterations in the *TA* muscle but not in the *Soleus* muscle

To assess the effect of hypoxaemia on the regeneration potential of the two muscles, we investigated the effect of SIH on myogenic marker expression. Indeed, the myogenic process involves SCs, which are the main mediators of muscle regeneration and are characterised by the expression of specific myogenic factors within well-established expression windows. *Pax7* (encoding PAX7) is a marker of quiescent SCs while *Myf5* (encoding MYF5), *Myod1* (encoding MYOD1) and *Myog* (encoding Myogenin) are commonly considered as markers of specific differentiation stages [53]. Indeed, *Myf5* is expressed by activated SCs, *Myod1* is expressed at the myoblast stage, and finally *Myog* at the myocyte stage, inducing fusion into myotubes [54].

**Fig. 6:**
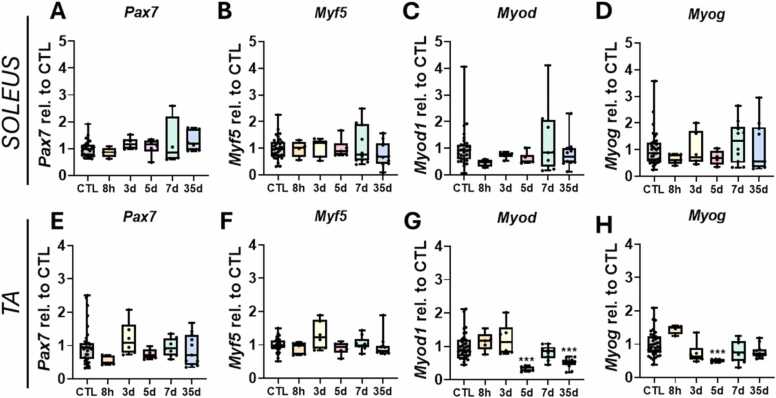
Effect of SIH exposure on myogenic marker expression in *Soleus* and *TA* muscles over time. (A-D) RT-qPCR were performed on the *Soleus* muscle. Gene expression was analysed by using the ΔΔCt method (housekeeping gene: *Rplp0*; data normalised to CTL). (A) *Pax7* (encoding PAX7) expression in the *Soleus* muscle; One-Way ANOVA: NS (CTL: n = 4–12; SIH: n = 5–12). (B) *Myf5* (encoding MYF5) expression in the *Soleus* muscle; One-Way ANOVA: NS (CTL: n = 4–12; SIH: n = 5–12). (C) *Myod1* (encoding MYOD1) expression in the *Soleus* muscle; One-Way ANOVA: NS (CTL: n = 4–12; SIH: n = 5–12). (D) *Myog* (encoding Myogenin) expression in the *Soleus* muscle; One-Way ANOVA: NS (CTL: n = 4–12; SIH: n = 5–12). (E-H) RT-qPCR were performed on the *TA* muscle. Gene expression was analysed by using the ΔΔCt method (housekeeping gene: *Rplp0*; data normalised to CTL). (E) *Pax7* (encoding PAX7) expression in the *TA* muscle; One-Way ANOVA: NS (CTL: n = 4–12; SIH: n = 5–12). (F) *Myf5* (encoding MYF5) expression in the *TA* muscle; One-Way ANOVA: NS (CTL: n = 4–12; SIH: n = 5–12). (G) *Myod1* (encoding MYOD1) expression in the *TA* muscle; One-Way ANOVA: ***: p < 0.001, 5d and 35d vs CTL (CTL: n = 4–12; SIH: n = 5–12). **(H)***Myog* (encoding Myogenin) expression in the *TA* muscle; One-Way ANOVA: ***: p < 0.001, 5d vs CTL (CTL: n = 4–12; SIH: n = 5–12).

Regarding the *Soleus* muscle, we observed no effect of SIH on the expression of these different markers when compared to the CTL group (Fig. 6A-D).

In the *TA* muscle, no change was observed regarding the expression of *Pax7* and *Myf5* (Fig. 6E-F). However, a decrease in *Myod1* and *Myog* expression was evidenced at 5 days of exposure to SIH (Fig. 6G-H). This decrease was also highlighted at 35 days of exposure for *Myog* expression when compared to the CTL group (Fig. 6G). Our results suggest an alteration of myogenic marker expression over time in the *TA* muscle, which was not observed in the *Soleus* muscle.

## Discussion

Numerous animal studies have investigated the effects of chronic hypoxaemia at the muscle level [16], [37], [49], [55]. However, the impact of episodic hypoxaemia, which is found in many respiratory insufficiencies such as COPD, is rarely studied. Indeed, while continuous hypoxaemia seems to be a main characteristic observed in patients with advanced-stage COPD [56], [57], a subgroup of patients presents episodes of desaturation and reoxygenation during sleep or exercise training [58], [59]. Our murine SIH model was designed to more closely mimic the sustained intermittent hypoxaemia found in this pathological condition. A period of acclimatisation of the mice to the experimental environment helped to minimise body weight loss. Unlike some studies that chose to carry out an acclimatisation week to hypoxaemia before the start of the protocol to reduce effects on body weight and food intake [37], we chose to avoid acclimating mice to hypoxia before the beginning of the protocol, but only to the environment to which the animals would be exposed. Furthermore, this acclimatisation period allowed the mice to get used to food restriction (8 h/day). As a result, we did not observe any body weight loss during the acclimatisation week, nor during the exposure to SIH, compared to the CTL group. The exposure device was also optimised to limit movement restriction compared to our previous study, which was not focused on skeletal muscle [60]. This optimisation, using large cages where the mice were free to move, prevented the increase in cortisol levels observed when mice were kept in smaller cages, indicating a reduction of stress (Supplemental Figure S3). This feature is important for the interpretation of our results since movement restriction could also be responsible for disuse muscle atrophy [61]. While we did not perform quantitative recording of locomotor activity during exposure, we observed that SIH and control animals exhibited a similar behavior consistent with their normal activity pattern during the resting period (light phase). An unenriched antioxidant diet was also used during the protocol to minimise the naturally occurring antioxidants in regular food that could interfere with the muscle-level signalisation and protect skeletal muscle from the hypoxia-induced alterations [17].

SIH led to an increase in haematocrit. This reflects the EPO synthesis at the renal level, induced by HIF-1, resulting in secondary erythrocytosis [60], [62], [63], [64], [65]. This elevation was also observed in numerous studies following exposure to hypoxia [60], [66], [67]. The latency of the increase (from 5 days) can be explained by the time required for the production and maturation of sufficient new red blood cells to detect an increase [68], [69]. This increase in red blood count evidenced that hypoxaemia was indeed induced in mice by our model of SIH.

### SIH leads to distinct morphological and metabolic effects depending on the muscle fibre composition

We chose to analyse *TA* and *Soleus* muscles in this study due to their distinct metabolic and functional properties, allowing to investigate muscle-specific responses to SIH. The *Soleus* is an oxidative muscle, whereas the *TA* is a mainly glycolytic muscle that is highly responsive to hypoxic stress and widely used in experimental models. We also have to mention that in rodents, the *TA* muscle exhibits regional heterogeneity, with the presence of oxidative fibres preferentially located in a defined area, as shown in Supplemental Fig. S6 [70].

In our SIH model, we observed a hypertrophy of type I myofibres in the *Soleus* muscle under hypoxic conditions. Such a hypertrophic effect of hypoxia has already been described by using models *in vitro*. Indeed, while severe hypoxia (5% O₂) inhibited the muscle cell differentiation process, moderated hypoxia (10–15% O₂) led to muscle C2C12 cell hypertrophy [71]. In our study, the hypertrophy was only observed in the slow-twitch type I (oxidative) fibres of the *Soleus* muscle, but not in the fast-twitch *TA* muscle. These results are consistent with literature data reporting that myofibres respond differently to hypoxaemia depending on their predominant metabolism [49]. Indeed, slow fibres, which contain a larger number of mitochondria, primarily function through oxidative phosphorylation [20]. This type of metabolism makes them more vulnerable to oxygen deficiency [21], [22]. In contrast, fast fibres function mainly through the glycolytic pathway [20]. The *Soleus* muscle, rich in type I (slow) and IIa (slow intermediate) fibres, was reported to exhibit a higher basal expression of HIF-1 markers and a greater activation of compensatory mechanisms in response to hypoxia than glycolytic muscles [25]. Contrary to the results obtained in our study, the literature mainly reports an atrophic effect on muscles exposed to hypoxic conditions [20], [55], [72]. To our knowledge, very few studies have conducted fibre type-specific morphometric analyses in mice exposed to intermittent hypoxaemia, and no previous study has performed this comparison in the context of the SIH pattern which mimics a periodic and moderated hypoxemia. Moreover, studies primarily reported the effects of hypoxaemia on glycolytic muscles, which have a lower capacity to adapt to oxygen variations [73], due to their lower mitochondrial content, reduced vascularization and low levels of antioxidant enzymes [27], [74]. We can thus hypothesise that type I fibre hypertrophy at late timepoint reflects a compensatory response to SIH which does not occur—or at least occurs less efficiently—in fast-type fibres.

SIH mice did not exhibit a significant variation of myofibre type proportion, neither in the *Soleus* muscle nor in the *TA*. In this regard, discrepancies remain in the literature depending on the hypoxemic pattern. Indeed, in a model of continuous normobaric hypoxaemia (FiO₂: 10%, 24 h/day for 4 weeks), mice presented a shift in myofibre composition in the *Gastrocnemius* (mixed fibre-type muscle) and *Soleus* muscles, characterised by a transition from type IIx and IIb fibres in favour of type IIa fibres, while type I fibres remained unaffected [30]. In another study performed in a rat model, chronic hypoxaemia (FiO₂: 12.5%, 24 h/24 for 10 weeks) was shown to affect locomotor muscles, via a slow-to-fast myofibre shift associated with oxidative stress [33]. In our study, we cannot exclude an ongoing myofibre switch in SIH conditions. Indeed, if myofibres are commonly classified into major subtypes, they form a highly dynamic continuum where MyHC expression can adapt in response to different stimuli [75]. To confirm this hypothesis, a quantification of hybrid I/IIa and IIa/IIb myofibres would be informative to confirm such a myofibre-type transition.

The response to hypoxia also diverges with respect to HIF-1 target genes, particularly when focusing on genes involved in metabolism. We chose to evaluate the expression of well-known HIF-1 target genes, which are widely used as functional readouts of HIF-1 transcriptional activity [76]. *Pdk1*, the gene encoding the PDK1 metabolic switch regulator through inhibition of pyruvate dehydrogenase, would promote glycolysis and thereby decrease oxygen consumption in the muscle [77]. Consistent with our results obtained in the SIH model, its expression was shown to be increased in the *Soleus* in response to acute hypoxia conditions, but not in the *TA* [29]. This effect could be explained by the predominantly glycolytic basal metabolism of the *TA*, which is less dependent on oxygenation levels [29]. *Vegfa*, whose expression increases in response to hypoxia *via* HIF-1, plays a key role in angiogenesis to maintain sufficient oxygen supply to tissues [78]. *Ddit4* is expressed in cellular stress conditions, such as hypoxia, and leads to mTORC inhibition, suppressing protein synthesis [51]*.* Under SIH conditions, *Vegfa* and *Ddit4* were upregulated in both *Soleus* and *TA* muscles. However, the magnitude of the increase was greater in the *Soleus*, likely due to its higher oxygen dependence [29]. It is also important to note that differences in resting blood flow between the *TA* and the *Soleus* muscles exist, as well as within *TA* subregions [79]. Here, we observed, in the entire muscle, that *Vegfa* expression, a key hypoxia-response gene involved in angiogenesis, displayed a higher increase in the *Soleus* compared to the *TA* muscle upon SIH exposure. This is consistent with the notion that muscles with higher oxidative capacity and blood flow exhibit a more pronounced adaptive response to hypoxaemia.

Regarding muscle atrophy observed in COPD patients [80] it is important to emphasize that this muscle mass loss is multifactorial and cannot be explained only by SIH. Indeed, reduced exercise capacity, physical inactivity, systemic inflammation, and nutritional alterations all contribute to the atrophic phenotype described in patients [3]. In the present study, we aimed to specifically isolate one hypoxemic component of the COPD pathophysiology using the SIH model, thereby reproducing only one aspect of the disease in a reductionist approach. SIH alone may be insufficient to induce muscle wasting in the absence of other systemic and behavioral factors mentioned here above. Furthermore, the intermittent, slow cycling, sustained, and moderate pattern of the hypoxic exposure reflects an intermediate stage of the disease rather than advanced COPD, where muscle atrophy is more pronounced [80]. Therefore, while this model allows the characterization of specific effects of the SIH pattern, it does not fully represent the complex pathophysiological phenotype of COPD patients.

### SIH modulates muscle mass regulators in a muscle-type-dependent manner

Myostatin is a well-known negative regulator of muscle mass, produced by skeletal muscle and acting on its receptor ActRIIB [81], [82]. Myostatin specifically inhibits the phosphorylation of Akt and induces the expression of atrophy-related genes via FOXO transcription factors, leading to muscle atrophy [81]. Myostatin level was shown to be increased at mRNA and protein levels in the *Soleus* and *Gastrocnemius* muscles of rats exposed to hypobaric hypoxaemia (simulated altitude of 4500 m for 5 weeks) [22]. Myostatin serum levels are also increased in patients with COPD [23], even if the role of hypoxemia in this effect remains to be demonstrated, given confounding factors. Here, our data revealed that SIH is associated with a decrease in myostatin plasma level after 35 days, compared to the control group. This might potentially be attributed to the early decrease in *Mstn* expression in the *TA* fast-twitch muscle, one of the main sites of myostatin production [83]. However, such a decrease was only observed at the mRNA level after 5 days of SIH but was no longer observed at later timepoints. This plasmatic decrease in myostatin levels without change in the expression within muscles suggests the involvement of post-transcriptional and/or post-translational regulatory mechanisms. Myostatin is synthesized as an inactive pre-proprotein that undergoes several steps of proteolytic maturation before being secreted as an active form [84]. Variations in the activity of these proteases can influence the amount of circulating active myostatin independently of changes in mRNA levels. Moreover, circulating myostatin is strongly regulated, notably by the myostatin propeptide, follistatin, or GASP−1, which can modulate its bioavailability [85]. An increase in the amount of these inhibitors could thus contribute to the decrease in measured plasma levels. Myostatin is also produced by other tissues, such as muscles other than the ones studied in this work, as well as by adipose tissue, which can contribute to circulating levels. Differential regulation according to the tissues could explain the absence of local mRNA variation despite a systemic decrease in the amount of the protein. We should also note an increase of plasmatic myostatin over time in both the SIH and control groups, likely due to prolonged exposure to baseline experimental conditions, but without affecting SIH and control group comparability.

Regarding the expression of genes encoding myostatin (*Mstn)* and its receptor (*Acrv2b*), they have been reported to be predominantly expressed in fast-twitch type IIb fibres (IIb > IIx > IIa > I), which exhibit a larger cross-sectional area [82], [83], [86]. In our CTL group, similar results were observed for *Mstn* expression, which was higher in the *TA* muscle (fast-twitch) than in the *Soleus* muscle (slow-twitch). In contrast, the *Acvr2b* receptor appears to have the same expression level in both muscles under basal conditions. When comparing *Mstn* expression in the two muscles after 35 days of SIH exposure, we once again observed a higher *Mstn* expression in the *TA* compared to the *Soleus* muscle, this time accompanied by an increased *Acvr2b* receptor expression in the *Soleus* compared to the *TA*.

The decrease in plasma myostatin level at 35 days could, *per se,* supports the hypertrophic effects on type I fibres observed in the *Soleus* muscle of SIH mice [83], but concomitantly, an increase in *Acvr2b* expression was observed in the same muscle. This observation could reflect a compensatory mechanism in response to the decrease in circulating myostatin. Indeed, a reduction in the ligand could induce an up-regulation of its receptor to maintain tissue sensitivity to this signal [87]. Therefore, even if not investigated in our study, myostatin receptor protein level, location, and downstream pathways might be interesting to better decipher the relative sensitivity of *Soleus* and *TA* muscles to myostatin circulating levels.

Skeletal muscle adaptive response to hypoxaemia is often associated to modifications in the expression and/or activity of protein synthesis and degradation regulators [16], [17], [21]. However, this response is highly dependent on the exposure pattern used, the type of muscle studied, and the animal model employed [24], [49], [86]. In the SIH model, we observed muscle-specific effects when investigating the expression of genes encoding ubiquitin ligases Atrogin−1 (*Fbxo32)* and MURF1 (*Trim63)*. First, *Fbxo32* was investigated as it is known to be responsible for the degradation of protein components involved in protein synthesis (MYOD1, eIF3-f) [88]. The baseline *Fbxo32* expression appeared similar in the two muscles. However, upon exposure to hypoxaemia at an early time point (8 h), a higher expression was observed in the *TA* muscle when compared to the *Soleus*. This increase seems delayed in the *Soleus* muscle. This is consistent with other studies reporting an attenuated response in the *Soleus* muscle regarding *Fbxo32* expression due to its more oxidative basal metabolism [89]. On the other hand, *Trim63* was investigated as a marker of muscle contractile protein degradation (proteins involved in ATP generation and myofibrillar proteins) [88]. The expression of this gene significantly increased in the *TA* muscle at early timepoint, but not in the *Soleus* muscle. These data are in accordance with the hypertrophic phenotype observed in the *Soleus* muscle, but not in the *TA* muscle, where the early increase in the expression of both atrogenes supports an early activation of proteolysis in response to hypoxia, as often reported under these conditions [72], [90]. Indeed, mice exposed to normobaric hypoxia (FiO2 8%, 24 h/24) showed an increase in *Fbxo32* expression after 2 and 4 days of exposure in the *Gastrocnemius* muscle. This early degradation of contractile proteins via *Trim63* and protein synthesis factors via *Fbxo32* reinforces the lack of morphological effects observed at 35 days of SIH in the *TA* muscle.

### SIH differentially affects myogenic marker expression according to muscle type

In our model, the expression of myogenic markers appears altered in the *TA*, but not in the *Soleus* muscle. This result is consistent with a previous study showing that hypobaric hypoxaemia did not impact the protein levels of MYOD1 and MYOG in the *Soleus* muscle of rats when compared to normoxia [37]. Our results suggest an impairment of the regenerative capacity of the *TA* muscle of SIH mice, that have to be further confirmed in a lesional context. The fact that the *Soleus* muscle does not show any alteration in these markers indicates that the myogenic process was not affected in this muscle, suggesting that such an alteration would not limit its growth under hypoxemic conditions. On the contrary, the altered expression of myogenic markers observed in the *TA* muscle could potentially limit muscle growth [91], a result in accordance with the absence of muscle hypertrophy in this muscle upon hypoxaemia.

## Conclusion

In conclusion, we developed a murine model of Sustained Intermittent Hypoxaemia to mimic the hypoxemic component of COPD patients and better understand its specific effect on skeletal muscle. We highlighted that SIH effects are muscle-type specific. Notably, slow type-I *Soleus* fibres presented a hypertrophy at the late timepoint, together with an early upregulation of HIF-1 target genes, indicating a compensatory response to SIH. In the fast *TA* muscle, SIH did not impact myofibre CSA. Contrary to the *Soleus*, this muscle did not exhibit any increase in the expression the HIF-1 target gene *Pdk1*, suggesting distinct metabolic adaptations. The *TA* muscle was also characterized by an earlier and greater upregulation of two atrogenes, consistent with the absence of hypertrophy. Importantly, this muscle showed altered myogenic marker expression, suggesting an altered regenerative capacity that remains to be assessed in a lesional context. In addition, myostatin plasmatic level was reduced upon SIH and could potentially be involved in muscle-type-specific effects observed, given the differential receptor expression in both muscles at baseline and upon SIH. Further investigations are now needed, notably at the protein levels, to decipher pathways involved in the muscle-type-specific effects of SIH and, particularly, to determine molecular mechanisms underlying the differential adaptive responses depending on myofibre type.

## List of abbreviations

ACVR2B: Activin A Receptor Type IIB

Akt: Protein Kinase B

COPD: Chronic Obstructive Pulmonary Disease

CSA: Cross-Sectional Area

CTL: Control

DDIT4: DNA Damage-Inducible Transcript 4

EDL: Extensor Digitorum Longus

eIF3-f: Eukaryotic Initiation Factor 3 subunit f

ELISA: Enzyme-Linked Immunosorbent Assay

EPO: Erythropoietin

FBXO32: F-box Protein 32

FiO_2_: Fraction of Inspired Oxygen

FOXO: Forkhead box O

HIF1: Hypoxia-Inducible Factor 1

MSTN: Myostatin

mTORC: mTOR Complex

MYF5: Myogenic Factor 5

MyHC: Myosin Heavy Chain

MYOD1: Myogenic Differentiation 1

MYOG: Myogenin

NS: Non-Significant

NOTCH: Notch signaling pathway

OCT: Optimal Cutting Temperature compound

OxPhos: Oxidative Phosphorylation

PAX7: Paired Box protein 7

PBS: Phosphate-Buffered Saline

PDK1: Pyruvate Dehydrogenase Kinase 1

RT: Room Temperature

RTqPCR: Reverse Transcription Quantitative PCR

SCs: Satellite Cells

SIH: Sustained Intermittent Hypoxaemia

TA: *Tibialis Anterior*

TRIM63: Tripartite Motif Containing 63

VEGFA: Vascular Endothelial Growth Factor A

WNT: WNT signaling pathway – Wingless-related integration site signaling pathway

## Author’s contribution

L.P., A.L., C.M., A.T. conceived and designed the research; L.P. and V.J. performed the experiments; L.P. analyzed the data; L.P., C.M., and A.T. interpreted the results of experiments; L.P., C.M., and A.T. prepared figures; L.P., C.M., and A.T. drafted the manuscript. L.P., V.J., A.L., C.M., and A.T. approved this version of the manuscript.

## CRediT authorship contribution statement

**Alexandra TASSIN:** Writing – review & editing, Writing – original draft, Visualization, Validation, Supervision, Resources, Project administration, Methodology, Funding acquisition, Formal analysis, Data curation, Conceptualization. **Carine MICHIELS:** Writing – review & editing, Writing – original draft, Visualization, Validation, Supervision, Resources, Project administration, Methodology, Funding acquisition, Formal analysis, Data curation, Conceptualization. **Alexandre LEGRAND:** Visualization, Validation, Supervision, Conceptualization. **Vincianne JENART:** Investigation. **Paprzycki Lise:** Writing – review & editing, Writing – original draft, Visualization, Validation, Resources, Project administration, Methodology, Investigation, Funding acquisition, Formal analysis, Data curation, Conceptualization.

## Ethics approval

All procedures met the Belgian national standard requirements regarding animal care and were conducted in accordance with the Ethics and Welfare Committee of the University of Mons (reference number of the approved protocol LE026/01).

## Funding

L.P. holds a UMONS Research Council PhD fellowship and a UMONS-UNamur PhD grant. The authors acknowledge the funding from the Research Institute for Health Sciences and Technology, UMONS (Grant call 2023-2025) and the "Fondation Raoul Warocqué pour la recherche médicale en Hainaut" (Grant call 2025).

## Declaration of Competing Interest

The authors declare the following financial interests/personal relationships which may be considered as potential competing interests: Alexandra Tassin reports that financial support was provided by Fondation Raoul Warocque. Alexandra Tassin reports that financial support was provided by the Research Institute for Health Sciences and Technology (UMONS). Lise Paprzycki reports that financial support was provided by UMONS-UNAMUR Research Councils. Lise Paprzycki reports that financial support was provided by the UMONS Research Council. If there are other authors, they declare that they have no known competing financial interests or personal relationships that could have appeared to influence the work reported in this paper.

## Data Availability

All data supporting the findings of this study are included in the article and its supplementary information files.
