# Supplementary material for: Slow and fast locomotor muscle adaptations to sustained intermittent hypoxaemia
Source: J Physiol Sci. 2026 Jun 20;76(2):100084. doi: 10.1016/j.jphyss.2026.100084 (PMC13333354; doi:10.1016/j.jphyss.2026.100084)
Supplement: Supplementary file 1 — Supplementary material [file mmc1.docx]

Supplementary data


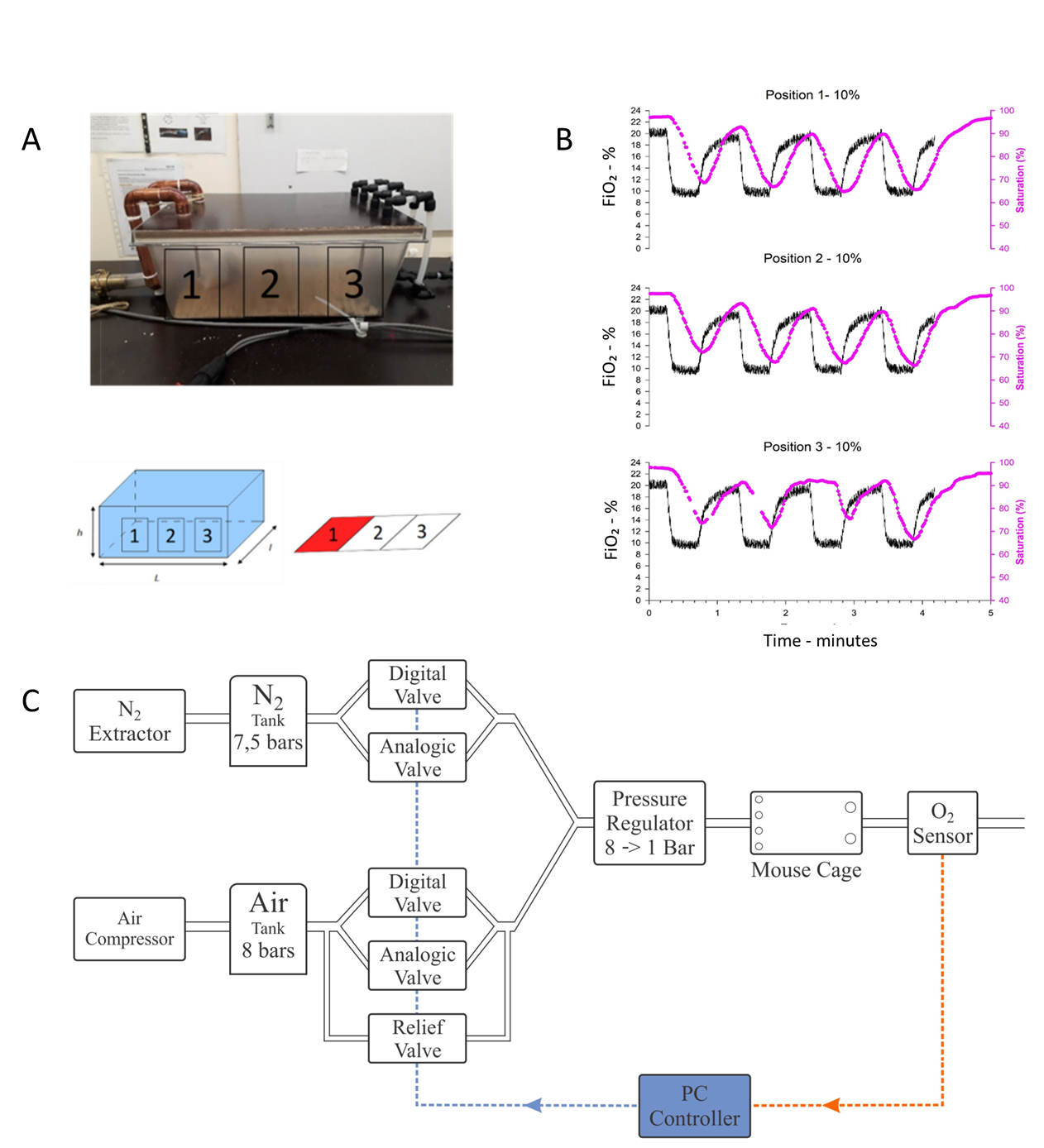


**Figure S1: Evaluation of Fraction of inspired Oxygen (FiO₂) and haemoglobin oxygen saturation (SaO2) of mice in the device allowing for exposure to hypoxic conditions. (A)** The cage (39 x 25 x 15cm) has been virtually divided into 3 areas. **(B)** FiO₂ (in black) was measured in these 3 areas by using an oxygen sensor. SaO_2_ (in purple) was assessed in 3 mice positioned in the 3 areas by transcutaneous measurements using an oximeter. Cycles of one minute were applied: a desaturation phase of 30 seconds (FiO₂ 10%) was followed by a reoxygenation phase of 30 seconds (FiO_2_ 21%); this cycle was repeated for a total of 4 minutes. No difference was observed according to the position within the cage. **(C)** Circuit diagram of the device.

**Table S2: primers used for RT-qPCR analyses**

| Target gene | Primers | Melting t° |
| --- | --- | --- |
| *Rplp0* | Forward: 5’-GGA-CCC-GAG-AAG-ACC-TCC-TT-3’  Reverse: 5’-GCA-CAT-CAC-TCA-GAA-TTT-CAA-TGG-3’ | 60°C |
| *Pdk1* | Forward: 5’-TGT-CTG-TCC-TGG-TGA-TTT-CG-3’  Reverse: 5’- TGG-CTA-TGA-GAA-CGC-TAG-GC-3’ | 62°C |
| *Ddit4* | Forward: 5’-GGC-CGG-AGG-AAG-ACT-CC-3’  Reverse: 5’-CAT-CAG-GTT-GGC-ACA-CAG-G-3’ | 60°C |
| *Vegfa* | Forward: 5’-AAC-GAT-GAA-GCC-CTG-GAG-TG-3’  Reverse: 5’-GCT-GGC-TTT-GGT-GAG-GTT-TG-3’ | 62°C |
| *Mstn* | Forward: 5’-ATT-ATC-ACG-CTA-CCA-CGG-AAA-C-3’  Reverse: 5’-CCA-TCC-GCT-TGC-ATT-AGA-AAG-3’ | 60°C |
| *Acvr2b* | Forward: 5’-CTT-CTC-TGG-GGA-TCG-CTG-T-3’  Reverse: 5’-TGG-CGT-TGT-AGT-AGA-TGC-AC-3’ | 60°C |
| *Fbxo32* | Forward: 5’-GCA-AAC-ACT-GCC-ACA-TTC-TCT-C-3’  Reverse: 5’-CTT-GAG-GGG-AAA-GTG-AGA-CG-3’ | 60°C |
| *Trim63* | Forward: 5’-AGT-GTC-CAT-GTC-TGG-AGG-TCG-TTT-3’  Reverse: 5’-ACT-GGA-GCA-CTC-CTG-CTT-GTA-GAT-3’ | 60°C |
| *Pax7* | Forward: 5’-CAT-GAA-CCC-TGT-CAG-CAA-TG-3’  Reverse: 5’-CAC-TCG-GGT-TGC-TAA-GGA-TG-3’ | 62°C |
| *Myf5* | Forward: 5’-TGA-GGG-AAC-AGG-TGG-AGA-AC-3’  Reverse: 5’-AGC-TGG-ACA-CGG-AGC-TTT-TA-3’ | 62°C |
| *Myod1* | Forward: 5’-CCA-CTC-CGG-GAC-ATA-GAC-TTG-3’  Reverse: 5’-AAA-AGC-GCA-GGT-CTG-GTG-AG-3’ | 62°C |
| *Myog* | Forward: 5’-GAG-ACA-TCC-CCC-TAT-TTC-TAC-CA-3’  Reverse: 5’-GCT-CAG-TCC-GCT-CAT-AGC-C-3’ | 60°C |


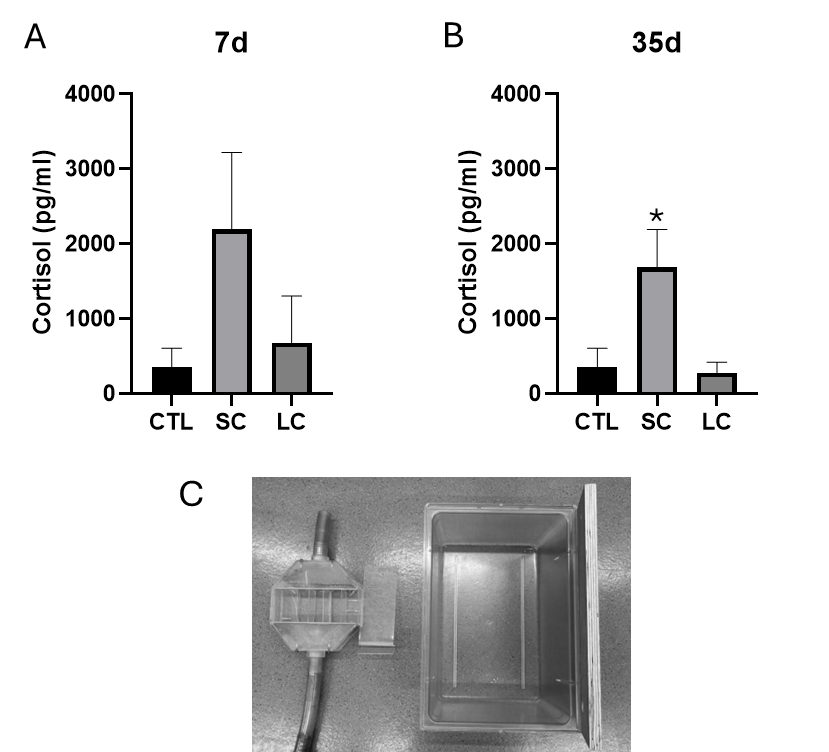


**Figure S3: Cortisol levels compared in small cages and large cages.** Plasma cortisol levels were assessed in mice submitted to SIH in small cages (SC) and large cages (LC) in comparison to mice kept in a normal atmosphere (CTL). **(A)** After 7 days of exposure; One-Way ANOVA: NS (n=5). **(B)** After 35 days of exposure; One-Way ANOVA: *p < 0.05, SC vs CTL (n=5). **(C)** Representations of small cages (left) and large cages (right).


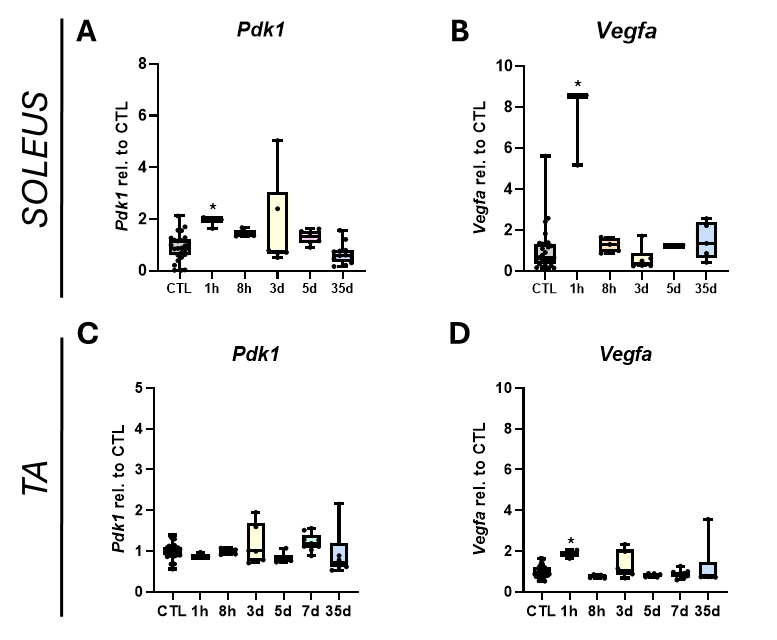


**Figure S4: Effects of SIH exposure on the expression of HIF1 target genes and modulators of cellular response to hypoxia in the *Soleus* (A-B) and the *TA* (C-D) muscles over time. (A-B)** RT-qPCR were performed on the *Soleus* muscle. Gene expression was analysed by using the ΔΔCt method (housekeeping gene: *Rplp0*; data normalized to CTL). **(A)** *Pdk1* (encoding PDK1) expression; One-Way ANOVA: *p < 0.05 vs CTL. **(B)** *Vegfa* (encoding VEGF) expression; One-Way ANOVA: *p < 0.05 vs CTL. **(C-D)** RT-qPCR were performed on the *Tibialis Anterior* (*TA)* muscle. Gene expression was analysed by using the ΔΔCt method (housekeeping gene: *Rplp0*; data normalised to CTL). **(C)** *Pdk1* (encoding PDK1) expression; One-Way ANOVA: NS. **(D)** *Vegfa* (encoding VEGF) expression; One-Way ANOVA: *p < 0.05 vs 8h.


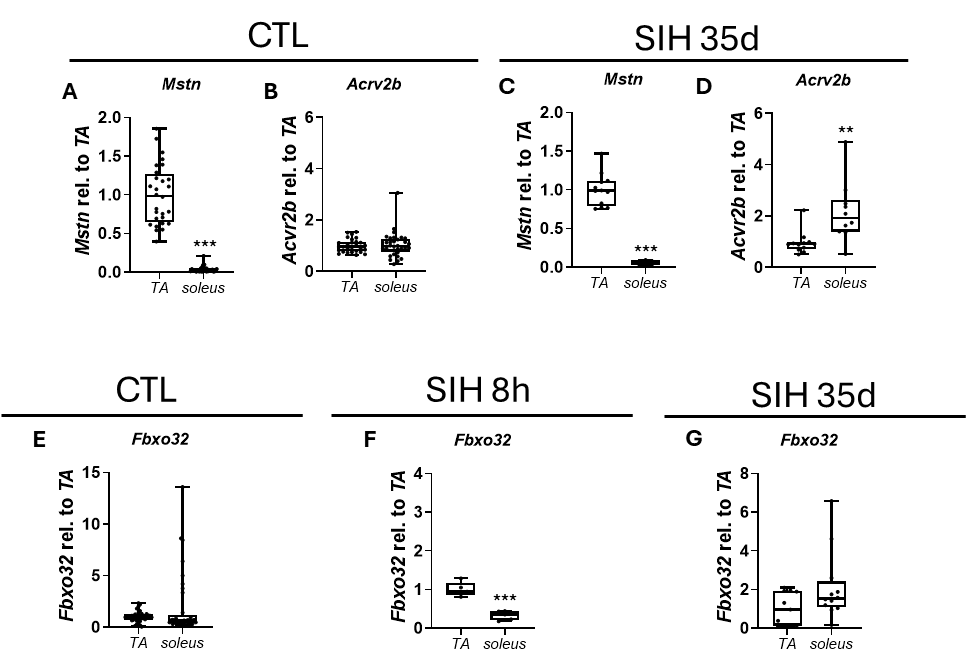


**Figure S5: Comparison of the expression of genes encoding protein degradation regulators in the *Soleus* and *TA* muscles under CTL or SIH conditions. (A-G)** RT-qPCR were performed on the *Soleus* and *TA* muscles. Gene expression was analysed using the ΔΔCt method (housekeeping gene: *Rplp0*; data normalised to TA). **(A)** *Mstn* (encoding Myostatin) expression in the *TA* vs *Soleus* muscle in the CTL mice; Mann-Whitney Rank Sum test: ***: p < 0.001, *Soleus* vs *TA* (CTL: n=38; SIH: n=39). **(B)** *Acvr2b* (encoding ActRIIB) expression in the *TA* vs *Soleus* muscle in the CTL mice; Mann-Whitney Rank Sum test: NS (CTL: n=38; SIH: n=39). **(C)** *Mstn* (encoding Myostatin) expression in the *TA* vs *Soleus* muscle in the SIH group; Mann-Whitney Rank Sum test: ***: p < 0.001, *Soleus* vs *TA* (CTL: n=12; SIH: n=12). **(D)** *Acvr2b* (encoding ActRIIB) expression in the *TA* vs *Soleus* muscle in the SIH group; Mann-Whitney Rank Sum test: **: p < 0.01, *Soleus* vs *TA* (CTL: n=12; SIH: n=12).  **(E)** *Fbxo32* (encoding Atrogin-1) expression in the *TA* vs *Soleus* muscle in the CTL group (Mann-Whitney Rank Sum test: NS (CTL: n=38; SIH: n=39)), **(F)** in the SIH 8h group (Mann-Whitney Rank Sum test: ***: p < 0.001, *Soleus* vs *TA* (CTL: n=4; SIH: n=5)) and **(G)** in the SIH 35d group (Mann-Whitney Rank Sum test: NS (CTL: n=12; SIH: n=12)).


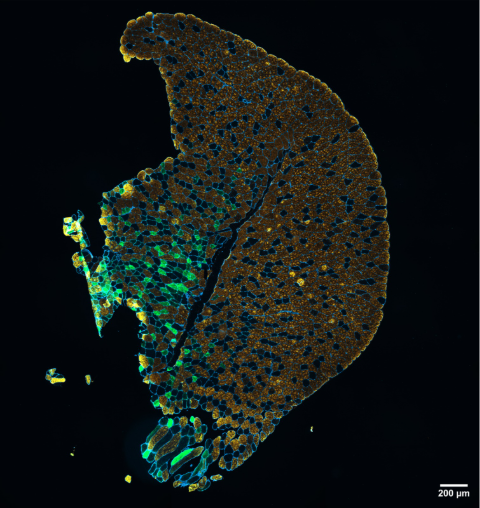


**Figure S6: Myofiber-type proportion analysis on an entire transverse section of the *Tibialis Anterior* (TA) muscle: representative field.** Muscle fibre types were identified using antibodies against MyHC isoforms: MyHC I (red), MyHC IIa (green), and MyHC IIb (orange). A specific spatial distribution pattern is observed, with MyHC IIa-positive fibres confined to a defined region of the muscle, while MyHC IIb-positive fibres are distributed throughout the section.
